# A novel mouse model for inhibition of DOHH-mediated hypusine modification reveals a crucial function in embryonic development, proliferation and oncogenic transformation

**DOI:** 10.1242/dmm.014449

**Published:** 2014-05-15

**Authors:** Henning Sievert, Nora Pällmann, Katharine K. Miller, Irm Hermans-Borgmeyer, Simone Venz, Ataman Sendoel, Michael Preukschas, Michaela Schweizer, Steffen Boettcher, P. Christoph Janiesch, Thomas Streichert, Reinhard Walther, Michael O. Hengartner, Markus G. Manz, Tim H. Brümmendorf, Carsten Bokemeyer, Melanie Braig, Joachim Hauber, Kent E. Duncan, Stefan Balabanov

**Affiliations:** 1 Department of Oncology, Hematology and Bone Marrow Transplantation with Section Pneumology, Hubertus Wald-Tumor Zentrum, University Hospital Eppendorf, 20246 Hamburg, Germany.; 2 Heinrich Pette Institute, Leibniz Institute for Experimental Virology, 20251 Hamburg, Germany.; 3 Center for Molecular Neurobiology (ZMNH), University Medical Center Hamburg-Eppendorf (UKE), 20251 Hamburg, Germany.; 4 Department of Medical Biochemistry and Molecular Biology, University of Greifswald, 17475 Greifswald, Germany.; 5 Institute of Molecular Life Sciences, University of Zurich, 8057 Zurich, Switzerland.; 6 Division of Hematology, University Hospital Zurich, 8091 Zurich, Switzerland.; 7 Department of Clinical Chemistry, University Hospital of Cologne, 50924 Cologne, Germany.; 8 Clinic for Internal Medicine IV, Hematology and Oncology, University Hospital of the RWTH Aachen, 52074 Aachen, Germany.

**Keywords:** Hypusine modification, Translational control, Cancer, Mouse models

## Abstract

The central importance of translational control by post-translational modification has spurred major interest in regulatory pathways that control translation. One such pathway uniquely adds hypusine to eukaryotic initiation factor 5A (eIF5A), and thereby affects protein synthesis and, subsequently, cellular proliferation through an unknown mechanism. Using a novel conditional knockout mouse model and a *Caenorhabditis elegans* knockout model, we found an evolutionarily conserved role for the DOHH-mediated second step of hypusine synthesis in early embryonic development. At the cellular level, we observed reduced proliferation and induction of senescence in 3T3 *Dohh*^−/−^ cells as well as reduced capability for malignant transformation. Furthermore, mass spectrometry showed that deletion of DOHH results in an unexpected complete loss of hypusine modification. Our results provide new biological insight into the physiological roles of the second step of the hypusination of eIF5A. Moreover, the conditional mouse model presented here provides a powerful tool for manipulating hypusine modification in a temporal and spatial manner, to analyse both how this unique modification normally functions *in vivo* as well as how it contributes to different pathological conditions.

## INTRODUCTION

The highly conserved and unique post-translational hypusine modification of a single cellular protein, the eukaryotic initiation factor 5A (eIF5A), represents an essential mechanism in the control of proliferation of eukaryotic cells (Cooper et al., 1982). This modification leads to the activation of eIF5A and is mediated by deoxyhypusine synthase (DHS), which catalyses the transfer of a 4-aminobutyl moiety of spermidine to the ε-amino group of Lys50 to form an intermediate residue, deoxyhypusine (Dhp50) (Park et al., 1981). Subsequently, deoxyhypusine hydroxylase (DOHH) mediates the formation of hypusine (Hyp50) by hydroxylation of the deoxyhypusine residue (Abbruzzese et al., 1986). A fundamental but yet unanswered question relates to whether there are functional differences between the unmodified eIF5A(Lys50), the intermediate deoxyhypusine-modified eIF5A(Dhp50) and the fully hypusine-modified eIF5A(Hyp50).

Hypusine modification occurs in two isoforms of human and rodent eIF5A. The eIF5A-1 isoform is expressed at high levels in all tissues, although the eIF5A-2 isoform is detectable only in some embryonic tissues, adult testis, central nervous system and cancer tissue (Clement et al., 2003; He et al., 2011; Jenkins et al., 2001; Zhu et al., 2011). Hypusine formation in eIF5A has been proposed to promote various cellular processes that potentially regulate proliferation, most notably mRNA translation (Henderson and Hershey, 2011; Saini et al., 2009) and nucleocytoplasmic transport of RNA (Lipowsky et al., 2000; Maier et al., 2010). Hypusine modification has also been implicated in numerous pathological conditions, including malignant transformation (Scuoppo et al., 2012; Zender et al., 2008), diabetes (Maier et al., 2010) and HIV infection (Bevec et al., 1996; Hauber et al., 2005). Although hypusine modification of eIF5A is highly conserved and essential for cell viability of all eukaryotes, its crucial cellular role in mammals remains unknown.

eIF5A was originally proposed to be an initiation factor because of its ability to stimulate methionyl-puromycin synthesis in cell-free translation extracts (Benne et al., 1978; Kemper et al., 1976). However, this has been questioned due to the lack of a significant effect of eIF5A on global protein synthesis in yeast (Kang and Hershey, 1994) or on the translation of native rabbit globin mRNA (Schreier et al., 1977). Recent studies have concluded that eIF5A functions primarily in translation elongation (Gregio et al., 2009; Saini et al., 2009). However, others have argued that the role of eIF5A in protein synthesis is likely to be confined to the formation of the first peptide bond during translation initiation (Henderson and Hershey, 2011). Given the modest effects on general protein synthesis, it has also been suggested that eIF5A could actually be important for the translation of a specific subset of mRNAs encoding, for example, cell cycle regulators (Hanauske-Abel et al., 1994; Kang and Hershey, 1994; Zuk and Jacobson, 1998). Encouraging reports in this direction have been described recently for yeast eIF5A and for the bacterial orthologue, the elongation factor P (EF-P). Like eIF5A, the activity of EF-P is regulated via a post-translational modification. In contrast to eIF5A, EF-P is not hypusinated, but lysinylated by YjeK and YjeA, and finally hydroxylated by YfcM (Bailly and de Crécy-Lagard, 2010; Navarre et al., 2010; Peil et al., 2012). Both eIF5A and EF-P have been shown to be particularly crucial for the synthesis of proteins containing consecutive prolines (Doerfel et al., 2013; Gutierrez et al., 2013; Ude et al., 2013).

**TRANSLATIONAL IMPACT**

**Clinical issue**

Post-translational modification (PTM) of proteins is critical for the regulation of a variety of cellular functions in normal and pathological conditions. Hypusine modification of the eukaryotic initiation factor 5A (eIF5A), a highly specific and conserved protein modification, has been linked to cancer, diabetes and infectious diseases. Although it is known that the PTM is an essential regulatory mechanism involved in eukaryotic cell proliferation, the specific role of hypusine modification in mammals has remained relatively elusive. Understanding the cellular function of this PTM in multicellular organisms is likely to provide insight into its pathological relevance in the context of disease.

**Results**

This study aimed to determine the importance of the second enzymatic step in hypusine modification in multicellular organisms. Using a gene targeting approach, the authors generated a mouse model allowing conditional knockout of deoxyhypusine hydroxylase (DOHH), the enzyme that catalyzes the second step in hypusine synthesis. Inactivation of both alleles of *DOHH* in this novel mouse model resulted in embryonic lethality. Furthermore, the authors established that loss of the *DOHH* homolog in *C. elegans* causes defects in early embryonic development. Analysis of the mutant phenotype in a mouse cell line revealed that loss of the enzyme leads to reduced cellular proliferation and a senescence-like phenotype. Mutant cells also demonstrated a reduced capacity for malignant transformation. Finally, application of mass spectrometry showed that deletion of DOHH causes a complete loss of hypusine modification.

**Implications and future directions**

This research established a novel mouse model that allows specific inhibition of the hypusine modification. The authors’ analysis of this model and validation in *C. elegans* provides new evidence that the DOHH-mediated second enzymatic step of hypusine synthesis is evolutionarily conserved and essential for development of higher eukaryotes. At the cellular level, the authors show that this PTM is required for proliferation of normal cells and affects the capacity of cells to undergo malignant transformation, which has implications for the relevance of hypusine modification in cancer. Importantly, this new mouse model for the conditional inhibition of the hypusine modification provides a tool to study the physiological and pathophysiological function of the PTM. In the long term, this model could advance the development of novel therapeutic approaches particularly in cancer and infectious diseases.

Temperature-sensitive *Saccharomyces cerevisiae* mutants revealed that the loss of either eIF5A or DHS function is lethal in yeast (Park et al., 1998; Sasaki et al., 1996; Schrader et al., 2006). Recently published constitutive knockout mouse models for eIF5A and DHS show embryonically lethal phenotypes, and thus support the vital function of the hypusine modification for the development of eukaryotic cells and organisms (Nishimura et al., 2012; Templin et al., 2011). For the second step of hypusine synthesis, catalysed by DOHH, the effect on growth and proliferation appears to be organism and cell-type specific. In yeast, DOHH knockout causes only a very mild growth phenotype (Park et al., 2006; Weir and Yaffe, 2004), implying that the second step of hypusination is dispensable for the essential function of eIF5A in this organism. In contrast, disruption of DOHH in *D. melanogaster* is lethal early in development (Patel et al., 2009), suggesting that the maturation of eIF5A(Dhp50) to eIF5A(Hyp50), catalysed by DOHH activity, might be crucial for the viability of higher eukaryotes. Thus, one can hypothesize that fully hypusine-modified eIF5A(Hyp50) plays a central role in multicellular organisms that eIF5A(Dhp50) cannot fulfil. Using gene targeting of *Dohh* in mice and *Caenorhabditis elegans*, we show that DOHH activity is crucial for mammalian development, as well as for proliferation and oncogenic transformation of a fibroblast cell line. Mass spectrometry analyses of eIF5A after depletion of DOHH revealed that hydroxylation of deoxyhypusine is essential for stabilization of hypusine. Moreover, we show that loss of DOHH in mammalian cells affects protein biosynthesis and provide strong evidence for a fundamentally important role of fully hypusinated eIF5A in mammals. Moreover, because our results constitute the first analysis of the role of DOHH in mammals, they have significant implications for targeted therapies aimed at inhibiting this enzyme.

## RESULTS

### Loss of *Dohh* causes lethality during embryonic mouse development

**Fig. 1.**
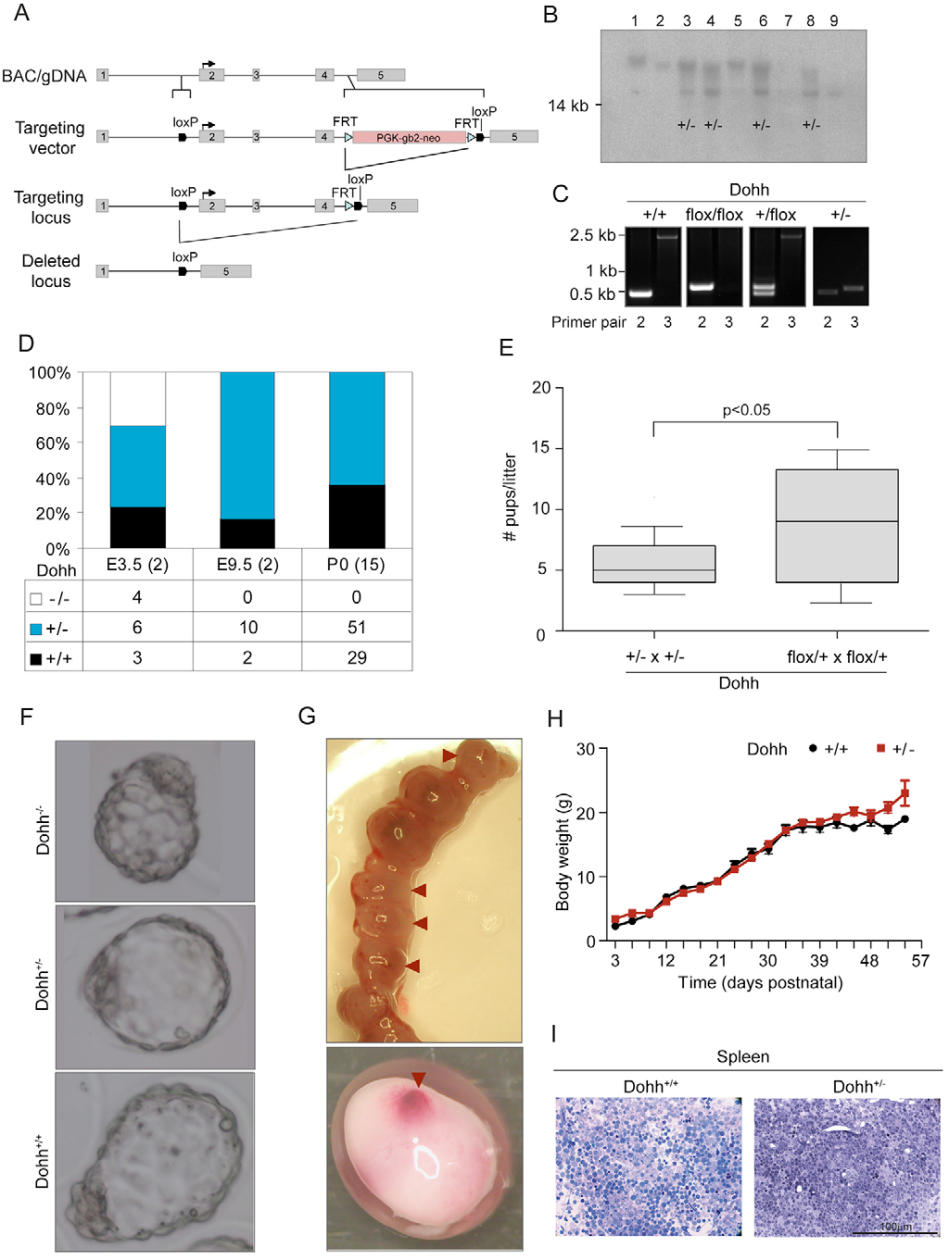
**Loss of both *Dohh* alleles elicits lethality during early embryonic development.** (A) Strategy for deletion of *Dohh* by introduction of *loxP* sites into introns 1–2 and 4–5 of *Dohh*. (B) Southern blot of ESC after recombination with the targeting construct. A transgenic (tg) 14-kb *Nde*I fragment is recognized after successful recombination in four out of nine clones. (C) Representative results for genotyping PCR for all the different genotypes. Primers are indicated in supplementary material Table S2. (D) Distribution of genotypes in litters of *Dohh*^+/−^ × *Dohh*^+/−^ matings. Numbers in parentheses indicate the number of examined litters. (E) Comparison of numbers of pups per litter in *Dohh*^+/−^ × *Dohh*^+/−^ against *Dohh*^+/flox^ × *Dohh*^+/flox^ matings. Significance has been determined using *t*-tests. (F) Representative blastocysts showing normal phenotype for wild-type, heterozygous and homozygous *Dohh* genotypes (E3.5). (G) The upper panel shows the uterus horn on E9.5 of a *Dohh*^+/−^ × *Dohh*^+/−^ mating. Arrowheads indicate implantation sites of smaller size than expected containing no embryos. The lower panel shows the uterine bead of a resorbed embryo after dissection of the outer muscular layers. (H) Body weight curves of mice of the *Dohh*^+/+^ and *Dohh*^+/−^ genotypes, showing no growth difference between both genotypes. (I) Morphological examination of the spleen of *Dohh*^+/−^ mice revealed diffuse infiltration compared with *Dohh*^+/+^ animals, probably with lymphoid cells. Representative micrographs out of three animals per genotype are shown.

To determine the molecular function of the second step of hypusine modification in mammals, we generated a mouse strain enabling conditional knockout of *Dohh* (B6.Dohh^tm1bal^). Inactivation of *Dohh* was achieved by using the Cre/loxP approach to target exons 2–4, which include both the *Dohh* start codon and three of the four His-Glu motifs essential for DOHH function (Kim et al., 2006) (Fig. 1A). Southern blot analysis and genotyping PCR confirmed correct recombination in embryonic stem cells (ESC; Fig. 1B) and accurate Cre-mediated *Dohh* deletion, respectively (Fig. 1C). To determine the specific role of eIF5A(Dhp50) in embryonic development, *Dohh* null allele (*Dohh*^+/−^) mice were generated by using CMV-Cre-deleter mice expressing Cre in early embryonic development (Schwenk et al., 1995). Heterozygous knockout mice (*Dohh*^+/−^) appeared normal with respect to genotype distribution. However, mating of *Dohh*^+/−^ mice produced no homozygous *Dohh*^−/−^ offspring (Fig. 1D). The observed *Dohh*^−/+^:*Dohh*^+/+^ ratio was almost 2:1, significantly varying from the expected Mendelian distribution of 1:2:1 (χ^2^-value=1.3×10^−6^) and indicating embryonic lethality of *Dohh*^−/−^ mice. Additionally, *Dohh*^−/+^ × *Dohh*^−/+^ litters were on average significantly smaller than *Dohh*^+/flox^ × *Dohh*^+/flox^ litters (Fig. 1E). In order to narrow down the exact time-point of lethality, we examined blastocysts and embryos at 3.5 and 9.5 days after conception (E3.5 and E9.5), respectively. Analysis of E3.5 blastocysts showed a normal Mendelian distribution of genotypes, including *Dohh*^−/−^ (Fig. 1D), and normal morphology (Fig. 1F). Genotyping of E9.5 embryos yielded results similar to those of postnatal offspring. However, implantation sites of significantly smaller size harbouring extra-embryonic tissue with no traceable residual embryonic tissue were found, indicating lethality before E9.5 (Fig. 1G). This indicates that loss of both *Dohh* alleles causes embryonic lethality arising after implantation into the uterus (E4.5) but well before E9.5. Compared with the strong effects of homozygous loss of *Dohh*, heterozygous mouse embryos survive gestation and are superficially indistinguishable from wild-type animals after birth. Over a period of about 50 days, we did not detect significant abnormalities, either in body weight (Fig. 1H) or in tissue morphology. It is worth noting that we observed a diffuse infiltration of the spleen with a homogenous, probably lymphoid, cell population in three *Dohh*^+/−^ animals, which was not present in any of the wild-type control mice (Fig. 1I). Because reports have linked hypusine modification to lymphoma development (Scuoppo et al., 2012), we examined the haematopoietic system (peripheral blood, bone marrow and spleen) of *Dohh*^+/−^ compared with *Dohh*^+/+^ mice in more detail. As depicted in supplementary material Figs S1 and S2, no significant haematological abnormalities appeared in young or adult *Dohh*^+/−^ animals compared with wild type.

### *dohh-1* is required for early embryonic development in *C. elegans*

**Fig. 2.**
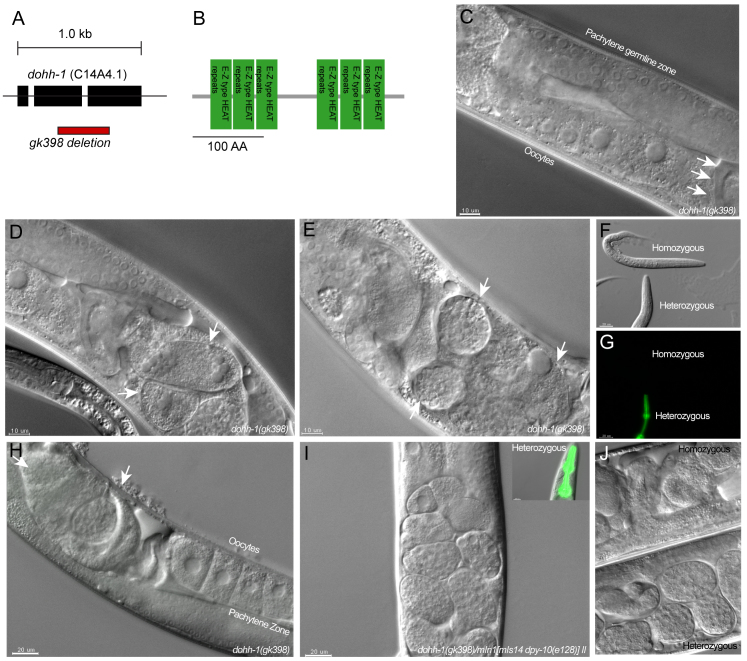
**Loss of *dohh-1* function in *C. elegans* leads to early embryonic lethality.** (A) Representation of the *C. elegans dohh-1* locus and the gk398 deletion allele. (B) *C. elegans* DOHH-1 protein contains 6 EZ-type HEAT repeat domains. (C–G) Differential interference contrast microscopy images (DIC) of *dohh-1* homozygous mutant animals show normal larval development (F–G). Adult animals exhibit wild-type germ lines (C), but defective embryos (arrows in D,E,H). (I–J) DIC of heterozygous *dohh-1(gk398)/mIn1[mIs14 dpy-10(e128)] II* animals.

Given the strong effects of a homozygous deletion of *Dohh* on early murine embryonic development, we pursued an additional approach to further characterize the role of DOHH in early development. *C. elegans* is a powerful model system for studying the function of genes during early embryonic development (Sönnichsen et al., 2005). The *C. elegans dohh-1* locus expresses a 33.2-kDa protein that shows 53.6% homology to mouse DOHH (Fig. 2A,B). The HEAT-repeats and iron-binding sites, both essential for enzyme function, exhibit a particularly high degree of homology. To determine the role of the DOHH homolog in *C. elegans* during development, we used the *dohh-1(gk398)* allele that deletes 371 bp of the *dohh-1* coding sequence. We found that maternally rescued homozygous *dohh-1* mutants completed larval (L1 to young adults) development normally, without any phenotypical abnormalities compared with heterozygous or wild-type animals (Fig. 2F,G). However, maternally rescued *dohh-1* homozygous mutants produced no progeny. Beginning from the one-cell stage, embryos exhibited strong abnormalities with development of sometimes multinucleated cells (Fig. 2D,E,H,J top). This resulted in accumulation of strongly altered, enlarged embryos in the uterus (Fig. 2H,J top) compared with heterozygous worms (Fig. 2I,J bottom). These data suggest that loss of *dohh-1* results in either a fertilization defect in the spermatheca or a defective cleavage process during blastocyst development.

### Loss of DOHH causes reduced proliferation and a senescence-like phenotype in fibroblasts

**Fig. 3.**
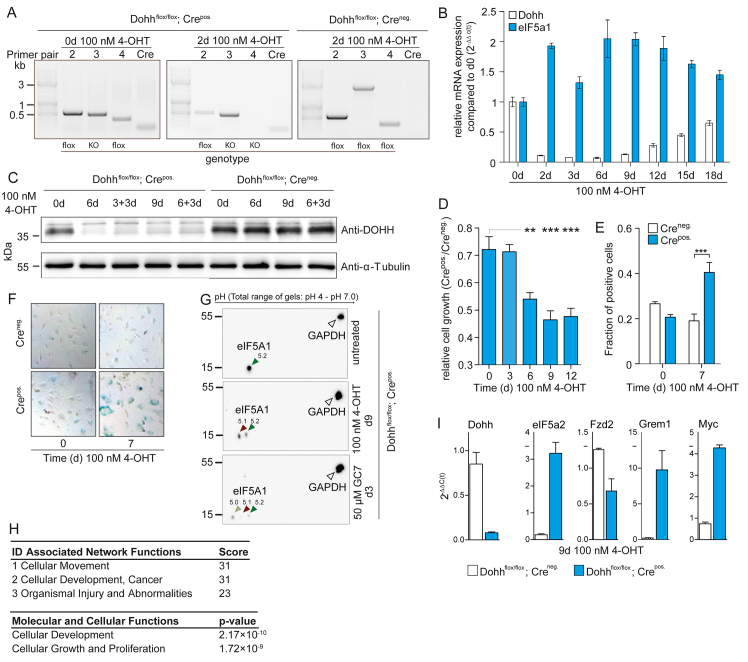
**Fibroblasts show reduced proliferation, a senescence-like phenotype and reduced hypusine modification upon complete loss of DOHH expression.** (A) Genotypes of 4-OHT-induced and uninduced cells, as well as a Cre-negative control cell line revealed the genomic deletion of both *Dohh* alleles in *Dohh*^flox/flox^; Cre^pos.^ 3T3 cells. Primers are indicated in supplementary material Table S2. (B) By using real-time RT-PCR, a decline of the *Dohh* transcript was observed in *Dohh*^flox/flox^; Cre^pos.^ cells relative to *Gapdh* and a Cre-negative control cell line (*Dohh*^flox/flox^; Cre^neg^). In parallel, expression of eIF5A was upregulated at the RNA level, most likely to compensate for the loss of DOHH. (C) Western blot analysis of DOHH in 4-OHT-induced and uninduced cells, as well as a Cre-negative control cell line revealed the reduction of DOHH protein expression in *Dohh*^flox/flox^; Cre^pos.^ 3T3 cells after deletion of *Dohh* compared with *Dohh*^flox/flox^; Cre^neg^ cells. Tubulin served as an internal standard. (D) Growth inhibition of *Dohh*^flox/flox^; Cre^pos.^ fibroblasts after 4-OHT induction, relative to a *Dohh*^flox/flox^; Cre^neg.^ control cell line. Level of significance was analysed using one-way ANOVA with Dunnett’s multiple comparison tests. (E) SA-β-Gal-positive cells after 1 week of 4-OHT induction, compared with a CreEsr1-negative control cell line. Level of significance was analysed using two-way ANOVA with Bonferroni post-tests. (F) Representative light microscopic images of cells of the experiments analysed in E, showing enlarged SA-β-Gal-positive cells after knock out of *Dohh*. (G) 2D western blots against eIF5A and GAPDH of fibroblast lysates treated as indicated on the panel depict a shift of a proportion of eIF5A from pH 5.2 (green arrow) to 5.1 (red arrow) after 4-OHT induction and a shift from pH 5.2 to 5.1 and 5.0 (yellow arrow), indicating a reduced hypusine modification. (H) Network function and gene ontology analysis of differentially expressed genes in Affymetrix microarrays after deletion of *Dohh* by 4-OHT in 3T3 *Dohh*^flox/flox^; Cre^pos.^ cells versus 3T3 *Dohh*^flox/flox^; Cre^neg.^ control cells. (I) Quantititive RT-PCR for some differentially expressed genes identified in the microarray analyses. qRT-PCR was performed in triplicates with *Gapdh* as a housekeeping control gene. ***P<0.001, **P<0.01.

To further examine possible mechanisms that lead to the observed strong phenotype *in vivo*, we established an immortalized 3T3 cell line based on mouse embryonic fibroblasts (MEFs) of floxed embryos, allowing for the inducible homozygous knockout of *Dohh*. Immortalized 3T3 cells were stably transfected with a retroviral pMSCV CreEsr1 expression plasmid or a negative control plasmid without the CreEsr1 insert. The extent of the 4-hydroxytamoxifen (4-OHT)-induced *Dohh* knockout was confirmed on DNA, RNA and protein levels (Fig. 3A–C). After knock out of *Dohh*, the cells showed a decrease in proliferation compared with control cells (Fig. 3D) and an increase in G2 phase of the cell cycle, but no overt cellular death (supplementary material Fig. S3). The anti-proliferative effects were less pronounced in unfloxed control cells (supplementary material Fig. S4) and could be partially compensated by retroviral Dohh overexpression (supplementary material Fig. S5). Of note, additional incubation of 3T3 *Dohh*^flox/flox^; Cre^pos.^ cells with 4-OHT and the DHS-inhibitor GC7 led to an even stronger reduction in cellular growth, most probably indicating an additive effect of DHS and DOHH inhibition on proliferation (supplementary material Fig. S6). Interestingly, knockout cells started to become rounder and flatter and increased their surface diameter by a factor of 5–10, showing a granular appearance in light microscopic analysis. As this phenotype strongly resembles cellular senescence, cells were stained for senescence-associated β-galactosidase (SA-β-gal). Indeed, knockout cells showed a significantly higher ratio of SA-β-gal-positive cells compared with the empty vector control (Fig. 3E,F), suggesting that proliferation of Dohh knockout cells is impaired by a senescent-like cell cycle arrest.

To ascertain whether the DOHH deficiency led to reduced hypusine modification of eIF5A(Dhp50), we examined the isoelectric points (pI) of modified and unmodified eIF5A isoforms using two-dimensional (2D) western blots, as described previously (Dyshlovoy et al., 2012). 2D western blots of lysates from DOHH-positive cells showed a single signal for eIF5A at a pI of 5.2, whereas a second signal appeared at a pI of 5.1 in DOHH-deficient cells, indicating the partial inhibition of hypusine modification (Fig. 3G). Control experiments using the DHS-inhibitor GC7 revealed an almost complete inhibition of the hypusine modification, marked by the appearance of a third, even more acidic, signal. To identify molecular pathways potentially involved in observed cellular effects, we performed a gene microarray analysis of 3T3 *Dohh*^flox/flox^; Cre^pos.^ cells after 4-OHT treatment compared with the control. Our results showed that a great number of genes showed altered expression levels after deletion of *Dohh* (supplementary material Table S1). Among these 464 upregulated and 791 downregulated genes, gene ontology (GO) and network analysis using Ingenuity software revealed that many were involved in cellular development, cancer and proliferation control (Fig. 3H). We confirmed modulation of expression after inhibition of hypusine modification for some genes by quantitative RT-PCR (Fig. 3I). Interestingly, we detected an upregulation of eIF5A2 as a consequence of deletion of *Dohh*. Furthermore, these analyses revealed a decreased expression of different members of the Wnt-pathway (e.g. *Fzd2*) and a strong upregulation of Myc (Fig. 3I).

### Hydroxylation of eIF5A by DOHH prevents reversibility of the DHS-mediated reaction

**Fig. 4.**
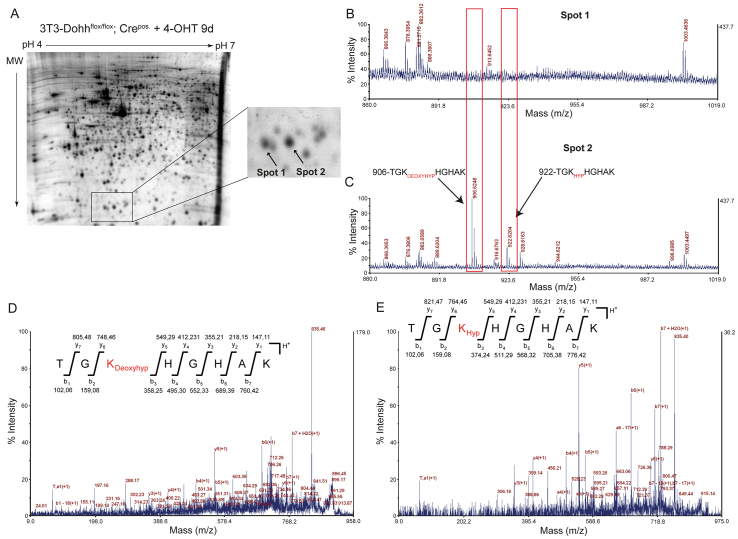
**Knock out of *Dohh* leads to loss of the entire hypusine modification.** (A) Representative 2D-PAGE of *Dohh*^flox/flox^; Cre^pos.^ 3T3 cells after treatment with 4-OHT and an enlarged region of the gel showing two spots for eIF5A at pH 5.2 and pH 5.1. (B) MS spectrum of eIF5A with an isoelectric point at 5.1 digested with LysC. Red frames highlight that neither the peptide for the deoxyhypusine-modified nor the peptide for hypusine-modified peptide appeared in the spectrum. (C) MS spectrum of eIF5A with an isoelectric point at 5.2 digested with LysC. In comparison with the more acidic isoform, both modified peptides (hypusine-modified and the deoxyhypusine-modified) are represented, with clear peaks at an *m/z* of 906.6248 and 922.6204, respectively. (D) Annotated y-and b-fragments of MS/MS spectra of the 906.6248 peptide shows the deoxyhypusine-modified lysine at position 50 of eIF5A. (E) Annotated y- and b-fragments of MS/MS spectra of the 922.6204 peptide shows the fully hypusine-modified eIF5A after hydroxylation by DOHH.

In the next set of experiments, we used mass spectrometry (MS) to characterize the effects of *Dohh* deletion on the modification of eIF5A at the molecular level. To isolate modified and unmodified eIF5A, we separated whole proteins lysates of 3T3 *Dohh*^flox/flox^; Cre^pos.^ cells after 9 days of 4-OHT treatment using large-scale 2D gel electrophoresis (Fig. 4A). We then picked two spots that, according to our previous research, corresponded to the unmodified (spot 1) and the modified eIF5A (spot 2), and digested both protein spots using endopeptidase LysC (Dyshlovoy et al., 2012). This enzyme provides the advantage of a hypusine-specific digest at Lys50 of eIF5A. If Lys50 is unmodified, LysC is able to cleave eIF5A on the carboxyl site of Lys50. In the presence of hypusine or deoxyhypusine, this site is blocked for LysC (Klier et al., 1995). In this case, the digest revealed a peptide of 906 Da (TGK_Deoxyhypusine_HGHAK) for the deoxyhypusine-containing eIF5A and a peptide of 922 Da (TGK_Hypusine_HGHAK) for the fully hypusinated protein, among others (Fig. 4C). In the presence of unmodified lysine, neither the 906-Da peptide nor the 922-Da peptide were detected (Fig. 4B). Further fragmentation clearly identified Lys50 as the site of modification responsible for the 16-Da mass difference between the 906-Da peptide (Fig. 4D) and the 922-Da peptide (Fig. 4E). The discrepancy of 16 Da is consistent with a loss of a hydroxyl group, indicating that after *Dohh* deletion, spot 2 consists of both the deoxyhypusine- and the hypusine-modified eIF5A. It is important to note that neither deoxyhypusine nor hypusine could be detected in spot 1 (Fig. 4B). Therefore, our data indicate that depletion of DOHH resulted in inhibition of hydroxylation and a subsequent loss of deoxyhypusine, leading to the appearance of spot 1. These observations suggest that the addition of a hydroxyl group to deoxyhypusine prevents the reversibility of the DHS reaction and stabilizes hypusine.

### Loss of *Dohh* affects H-Ras^V12^- and Myc-mediated transformation

**Fig. 5.**
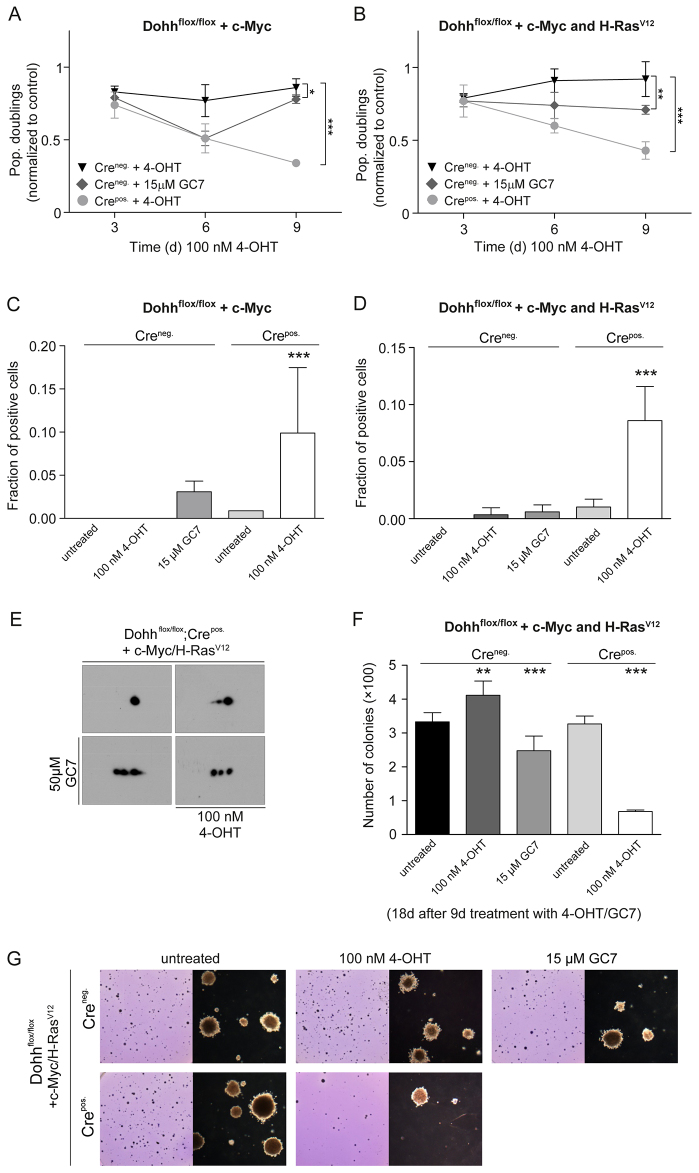
**DOHH activity affects malignant transformation of 3T3 fibroblasts.** (A) Population doublings of *Dohh*^flox/flox^; Cre^pos.^ or *Dohh*^flox/flox^; Cre^neg.^ 3T3 cells after treatment with 4-OHT or GC7, relative to untreated control cells. The 3T3 cells were retrovirally transduced with c-Myc. (B) Population doublings of *Dohh*^flox/flox^; Cre^pos.^ or *Dohh*^flox/flox^; Cre^neg.^ 3T3 cells after treatment with 4-OHT or GC7, relative to untreated control cells. Cells were transformed by subsequent retroviral transduction with c-Myc and H-Ras^V12^. (C,D) Quantification of senescence by SA-β-Gal assay in cells described in A and B after one week of 4-OHT or GC-7 treatment. (E) 2D western blots against eIF5A1 of H-Ras^V12^ and c-Myc transformed *Dohh*^flox/flox^; Cre^pos.^ cells treated with 4-OHT or GC7 or with both substances. (F) Number of colonies grown in soft agar after 9 days of pre-treatment with 4-OHT/GC7 in c-Myc and H-Ras^V12^ transformed *Dohh*^flox/flox^; Cre^pos.^ or *Dohh*^flox/flox^; Cre^neg.^ cells. GC7 was furthermore added to the top agar for the 18 days of incubation, whereas no extra 4-OHT was applied. (G) Representative photographs (left) and light microscopic images (right) of crystal violet stained and unstained colonies grown in soft agar of cells described in F. Level of significance was determined for all experiments using *t*-tests (***P<0.001, **P<0.01, *P<0.05). All analyses were performed at least in triplicate.

It has previously been shown that eIF5A and the DHS-mediated hypusine modification are linked to cancer (Guan et al., 2001; Preukschas et al., 2012; Tang et al., 2010; Zender et al., 2008). However, in the absence of genetic tools, it has previously not been possible to examine the impact of the second step of hypusine modification on malignant transformation. We therefore examined the effect of *Dohh* disruption on malignant transformation of 3T3 cells, using stable overexpression of oncogenic c-Myc or the combination of H-Ras^V12^ and c-Myc in *Dohh*^flox/flox^; CreEsr1^pos.^ 3T3 cells, which represent a widely used *in vitro* model to study effects on the malignant transformation of cells (Falcone et al., 1987). After induction of *Dohh* knockout in the presence of c-Myc or H-Ras^V12^ and c-Myc, cell proliferation decreased (Fig. 5A,B) and senescence induction increased (Fig. 5C,D), concordant with a reduction in hypusine-containing eIF5A in 2D western blots (Fig. 5E). This outcome was observed in partially transformed cells expressing c-Myc (Falcone et al., 1987), as well as fully transformed cells expressing H-Ras^V12^ and c-Myc. Interestingly, in an anchor-independent growth experiment, *Dohh*^−/−^ cells formed fewer transformed colonies. The number of visible colonies was reduced by ~80% compared with control cells (Fig. 5F), consisting mainly of small colonies (Fig. 5G). Treatment with 15 μM GC7, despite having a comparable impact on hypusine-containing eIF5A in 2D western blots (data not shown), had a less-pronounced effect on cell proliferation, induction of senescence and growth in soft agar. These results establish a relationship between DOHH-mediated hypusine modification and malignant transformation of mammalian cells.

### Loss of DOHH activity affects protein synthesis

**Fig. 6.**
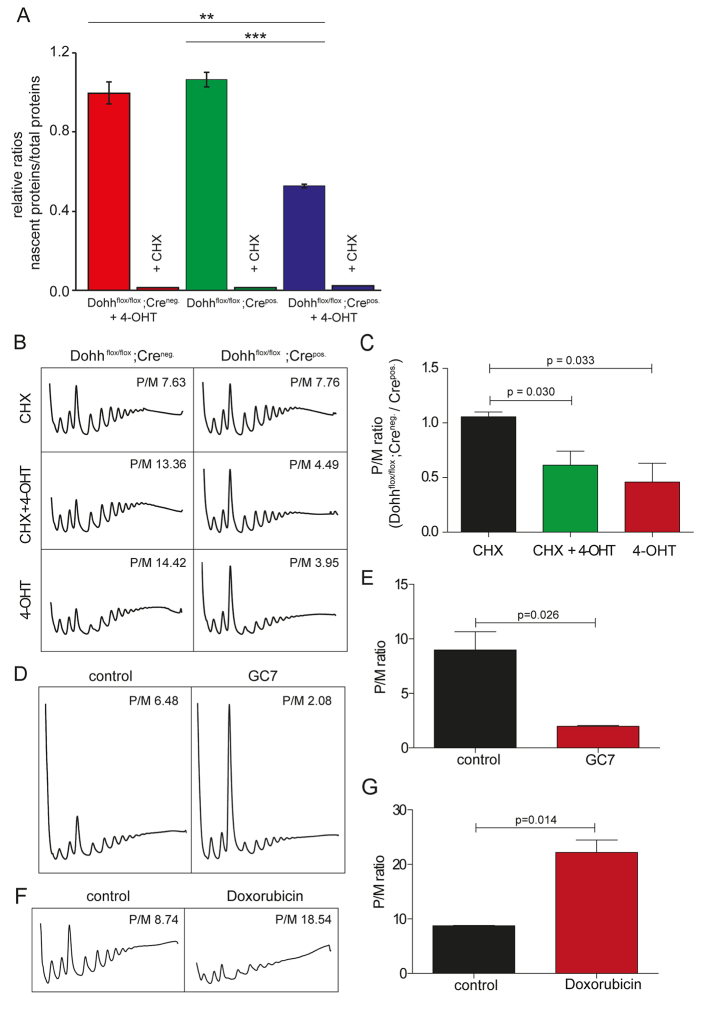
***Dohh* deletion impairs protein synthesis.** (A) *Dohh* depletion causes a reduction in the rate of protein synthesis. Cells were treated for 9 days with tamoxifen or were mock-treated as indicated. Newly synthesized proteins were labelled using bioorthogonal (‘Click’) chemistry. Label incorporated into newly synthesized proteins was normalized to total protein content to determine the relative rate of protein synthesis. Note the strong reduction in label incorporation in cells treated with CHX, which demonstrates that labelling depends on new protein synthesis and indicates the large dynamic range of the assay. (B) Polysome profile comparison of 3T3 cells with tamoxifen-inducible Cre removal of DOHH (*Dohh*^flox/flox^; Cre^pos.^) with those without (*Dohh*^flox/flox^; Cre^neg.^). Uninduced cells (CHX) show similar polysome profiles and P/M ratios. After removal of DOHH (*Dohh*^flox/flox^; Cre^pos.^: CHX/4-OHT and 4-OHT), profiles showed a clear increase in 80S, coupled with a mild reduction in polysomes, consistent with a translation initiation defect. (C) Ratios of P/M Cre^pos.^ to P/M Cre^neg^. for each condition described in A. Tamoxifen-induced cells show significantly reduced ratios in comparison with uninduced cells (CHX to CHX/4-OHT, *P*=0.03; CHX to 4-OHT, *P*=0.033; *n*=3; one-tailed, type 3 *t*-test). (D) Polysome profiles of 3T3 cells treated with GC7 to inhibit DHS activity. Profiles generated by GC7 treatment phenocopy those produced by removal of *Dohh*, but the effect is stronger. (E) Quantification of P/M ratios shows a significant difference between control and GC7-treated 3T3 cells (*P*=0.026; *n*=3; one-tailed, type 3 *t*-test). (F,G) Polysome profile and P/M ratio quantification for 3T3 cells treated with doxorubicin, an inhibitor of cell growth that does not directly interfere with translation. Note the absence of a *Dohh*-like initiation defect in doxorubicin-treated cells. Data are displayed as mean ± s.e.m. *P*-values were determined using Student’s unpaired *t*-test (**P<0.01, ***P<0.001).

eIF5A has been implicated in protein synthesis, but whether DOHH-mediated hydroxylation of hypusine is crucial for its role in this process in mammals has not yet been previously investigated. To address this question, we first performed metabolic labelling experiments to monitor new protein synthesis in the presence or absence of DOHH. We labelled newly synthesized proteins with non-radioactive methionine analogues and detected these using bioorthogonal (‘Click’) chemistry. This revealed a reduction in protein synthesis by roughly 50%, specifically in Dohh knockout cells (Fig. 6A). To gain insight into the mechanism, we used polysome profiling (Fig. 6B–G). As shown in Fig. 6B,C, a comparison of tamoxifen-induced 3T3-*Dohh*^flox/flox^; CreEsr1^pos.^ *Dohh* knockout cells and control cells revealed a decrease in the polysome/monosome (P/M) ratio in cells lacking DOHH, the hallmark of a translation initiation defect. A decreased P/M ratio relative to control cells was still observed even when the elongation inhibitor cycloheximide was omitted, indicating that cycloheximide treatment was not masking an elongation defect. Taken together, our metabolic labelling and polysome results indicate that both protein synthesis and translation initiation are compromised in mammalian cells lacking DOHH. Given the strong evidence from yeast that eIF5A plays a role in elongation, we presume that the apparent effect on translation initiation is likely to be an indirect effect (see Discussion). Treating cells with the DHS inhibitor GC7 also led to decreased P/M ratios (Fig. 6D,E), consistent with previous reports (Landau et al., 2010).

In principle, the effect on translation initiation observed with DOHH depletion could be a cause or consequence of altered cellular proliferation. To distinguish between these possibilities, we treated cells with doxorubicin, a proliferation inhibitor with no known direct effects on the mRNA translational machinery. As shown in Fig. 6F,G, polysome profiles from doxorubicin-treated cells revealed precisely the opposite effect to DOHH depletion and GC7 treatment. These results demonstrate that altered cellular proliferation does not necessarily mimic the effects of loss of DOHH function on protein synthesis in mammals.

## DISCUSSION

Hypusine modification has been implicated in the regulation of important cellular processes including translation, RNA metabolism, proliferation and apoptosis, in both health and certain diseases. However, the functional consequence of inhibiting the second step of this modification has not been extensively explored before. This study combines genetics and functional data from *C. elegans* with a new knockout mouse model to demonstrate that the DOHH-mediated step of hypusine modification has a pivotal function in murine and nematode development, in cell proliferation, malignant transformation and translation. Although studies in prokaryotes suggest that the polyamine network is functionally interchangeable and loss of an important enzyme can be mitigated by substitution (Tholl et al., 1998), the situation in eukaryotes seems to be more complex. Based on our results and recent studies on eIF5A and DHS (Enard et al., 2009; Hanazawa et al., 2004; Nishimura et al., 2012; Templin et al., 2011), it is reasonable to assume that in multicellular eukaryotes the elimination of any one of the members of the hypusine system is fatal regarding organism development. In this regard, it is important to note that gene inactivation studies on *Dohh* have revealed a mild effect on the phenotype in yeast (Park et al., 2006) and a more pronounced lethal phenotype in *Drosophila* (Patel et al., 2009). In line with the latter observation, we observed a strong effect of *Dohh* deletion on early embryonic development in *C. elegans* and mice. We also observed *Dohh* depletion to cause a strong alteration of the transcriptional activity of more than 1000 genes in 3T3 cells. How DOHH regulates gene transcription remains speculative, yet we assume that loss of DOHH affects eIF5A-dependent translation of proline-rich mRNAs comprising a variety of transcriptional regulators, therefore indirectly leading to broad changes in gene expression. It is worth noting that a significant subset of those genes are involved in developmental processes, thus explaining the observed developmental phenotypes. Our findings support the hypothesis that the second step of hypusine modification is particularly important for multicellular eukaryotes and expand the knowledge on the vital function of DOHH-mediated conversion of eIF5A(Dhp50) to eIF5A(Hyp50) in nematodes and mammals. These species-specific phenotypes can be due to different functions of eIF5A(Dhp50) and eIF5A(Hyp50) in single- versus multicellular organisms and might be based on conformational differences between eIF5A(Dhp50) and eIF5A(Hyp50) that lead to interactions with pathways specific to phylogenetically higher organisms. Interestingly, our studies in *C. elegans* revealed that the loss of *dohh-1* mirrors, at least partially, the previously observed effects of the deletion of the eIF5A-1 homologue *iff-2* on early embryonic development (Hanazawa et al., 2004). In analogy to this study, we detected strong effects on early embryonic development that might be due to effects on fertilization or on the cleavage process. It is worth noting that we did not observe the same phenotypical alterations in the germ line that were described for the *iff-1* isoform (Hanazawa et al., 2004). These observations suggest that, in *C. elegans*, the second step of hypusine modification is probably crucial for the function of *iff-2* but not for *iff-1* during embryonic development. Co-deletion of the *iff-1* or *iff-2* isoforms together with *dohh-1* would provide an appropriate strategy to further address that question.

A long-standing question with respect to hypusine modification is whether the hydroxylation of deoxyhypusine by DOHH is crucial for the stability of the final hypusine residue. Former *in vitro* studies have suggested that the hydroxyl residue prevents the reverse reaction of DHS, which is the rate-limiting reaction in hypusine modification (Wolff et al., 2007). Therefore, one important result of our study showed that deletion of *Dohh* results in an expected loss of the hydroxyl residue and in a surprising loss of the deoxyhypusine group on Lys50, leading to an accumulation of unmodified precursor eIF5A. These findings strongly suggest that, at least in mice, the *Dohh* deletion acts through an accumulation of both deoxyhypusine-modified and unmodified eIF5A. Therefore, the observed developmental and cellular defects in mice and worms can be based on an excess of intermediate or native eIF5A. As yeast cells are capable of surviving without DOHH and accumulating deoxyhypusine (Park et al., 2006), the physiological cellular conditions of single cell eukaryotes do not seem to provide an optimal environment for the reverse reaction of DHS. In yeast cells, accumulated deoxyhypusine-modified eIF5A might be sufficient to fulfil vital functions normally conducted by the hypusine-modified eIF5A, resulting in survival of *Dohh*-deleted strains. On-going studies using a conditional knockout mouse model for *Dhs* will help us to more clearly differentiate the impacts of DOHH and DHS function in higher eukaryotes.

Altered translational regulation is increasingly recognized to be an important factor in malignant transformation (Ruggero, 2013; Silvera et al., 2010). In this context, we and others have linked the hypusine modification system to various types of cancer (Preukschas et al., 2012; Scuoppo et al., 2012). In most of the studies, eIF5A is overexpressed in tumours and has been linked to patient prognosis in some types of cancer (Caraglia et al., 2013), although the impact of fully hypusine-modified eIF5A in cancer biology is still unknown. As inhibition of hypusine modification has been proposed to be a target for therapy of various types of cancer (Balabanov et al., 2007), we decided to test whether the specific deletion of *Dohh* affects the process of malignant transformation in cell culture. In this regard, our data support the assumption that the final step of hypusine modification of eIF5A is a crucial regulator of different characteristics of cancer cells. In line with other studies, our results suggest that inhibition of hypusine modification negatively regulates the proliferation of oncogene-transformed cells and reduces their capacity for anchor-independent growth. Post-translational modifications of other translational regulators have already been linked to the process of malignant transformation (Ruggero, 2013; Silvera et al., 2010), such as the regulation of phosphorylation of eIF2α, eIF4E and the 4E-binding proteins (Donzé et al., 1995; Furic et al., 2010; Wendel et al., 2007). Inhibition of translation has also been shown to increase the susceptibility of tumour cells to chemotherapy or targeted therapies (Balabanov et al., 2007; Bordeleau et al., 2008; Robert et al., 2009). Accordingly, the inhibition of hypusine-dependent translation of specific mRNA might have the potential to serve as a target for novel therapeutic intervention approaches in oncology. Due to the observed effects of *Dohh* depletion on normal cells *in vitro* and *in vivo*, future studies are required to identify hypusine-dependent pathways specific for cancer cells. In this context, it is worth highlighting a recent study that links the function of hypusine modification as a tumour suppressor in lymphoma development (Scuoppo et al., 2012). Here, Scuoppo and co-workers demonstrated that the shRNA-mediated reduction of eIF5A expression, or decreased hypusine modification through knockdown of DHS, augmented lymphoma development in the Eμ-myc mouse model. These results pointed out that the hypusine modification system has pleiotropic effects and can act either as promoter or suppressor of malignant transformation, depending on the cellular environment.

Interestingly, we observed an upregulation of eIF5A1 and eIF5A2 transcripts after deletion of *Dohh* (Fig. 3B,I), as was found in a recent *Drosophila melanogaster* study (Patel et al., 2009). However, the eIF5A1 protein expression level seems to be unaffected in semi-quantitative western blot analysis (supplementary material Fig. S7). Thus it seems that, at least on transcript level, a conserved positive feedback loop exists in eukaryotic cells, which might be activated to compensate for the decreased translational activity due to a loss of hypusine modification. In this respect it is important to note that we also observed a transcriptional upregulation of c-Myc. Because it has been shown that c-Myc directly regulates several components of the protein biosynthesis machinery, one can speculate that overexpression of c-Myc represents a further mechanism to compensate for reduced protein biosynthesis after the inhibition of hypusine modification (Barna et al., 2008; Grewal et al., 2005; van Riggelen et al., 2010). Interestingly, n-Myc controls spermidine synthesis via the ornithine decarboxylase (ODC) and ODC-activity antizyme (ODC-AZ), which consequently controls the spermidine supply for eIF5A (Geerts et al., 2010). Those findings, together with the observation that eIF5A gene expression is also subject to c-Myc regulation, might further support this notion (Coller et al., 2000).

We observed reduced protein synthesis and altered polysome profiles in cells lacking DOHH (Fig. 6A–C). An apparent effect on initiation was surprising because there is significant evidence from yeast for a primary role for hypusinated eIF5A in translation elongation (Saini et al., 2009) and knockdown of both eIF5A or DOHH in *Drosophila* cells led to polysome accumulation, consistent with an elongation defect (Patel et al., 2009). It was recently demonstrated in the lower eukaryote *Saccharomyces* that eIF5A particularly promotes the translation of polyproline regions (Gutierrez et al., 2013). This finding is in agreement with the reported activity of the eIF5A orthologue EF-P in bacteria (Doerfel et al., 2013; Ude et al., 2013). Thus, in our study using higher multicellular eukaryotes it is intriguing that loss of hypusine modification appears to affect initiation rather than elongation. However, the experiments presented here cannot distinguish between direct and indirect effects. In this context, it should be noted that eIF5A has also been reported to exert secondary activities in addition to promoting translation. In particular, eIF5A itself has RNA-binding properties (Xu and Chen, 2001) and at least two distinct cellular transcripts; the mRNAs encoding CD83 and iNOS, have been shown to be subject to eIF5A-dependent nucleocytoplasmic transport in mammalian cells (Kruse et al., 2000; Maier et al., 2010). Interestingly, although both transcripts are targets of eIF5A regulation, they do not encode typical polyproline stretches (three or more consecutive proline residues). It is therefore conceivable that, during evolution, eIF5A gained activities in addition to its primary role in translation elongation, such as the promotion of cytoplasmic accumulation of specific transcripts (Hauber, 2010). It is evident that such an activity would result in indirect effects on translation initiation, particularly in higher multicellular eukaryotes.

Another possible interpretation of our data is that loss of either DOHH or DHS function in mammals results in reduced translation elongation rates that lead to reduced levels of the proteins required for initiation, most likely containing polyproline regions (e.g. eIF-4E and eIF-5). Alternatively, inhibition of hypusine modification might perturb cellular carbon metabolism and thereby indirectly inhibit translation initiation (Castelli et al., 2011).

Of note, the observed *in vitro* effects in immortalized 3T3 cells were less pronounced than in the strong embryonic lethal phenotype. This might be due to activation of certain cellular pathways during the process of immortalization, rendering those cells less susceptible to a loss of DOHH. Therefore, further studies analysing the consequences of Dohh deletion in adult mice and primary non-immortalized cells harvested from different tissue (e.g. haematopoietic system and brain) need to be performed to elucidate the physiological functions of DOHH.

In conclusion, we have demonstrated that DOHH is crucial for early embryonic development of mice and *C. elegans*. Our studies suggest that inhibition of the final step of hypusine modification results in reduced efficiency of protein synthesis and establish this enzymatic reaction as essential for the cellular function of eIF5A and for the viability of mammalian cells. Furthermore, our work supports the hypothesis that the functional specificity of the highly conserved hypusine modification pathway phylogenetically evolved to an essential pathway from yeast to mammals.

## MATERIALS AND METHODS

### Animal studies

All animal experimental procedures were approved by the responsible Hamburg state authority according to German animal protection law.

### Cloning of targeting vector

The vector for targeting murine Dohh was cloned using the BAC Subcloning Kit as well as the Quick & Easy Conditional Knockout Kits (Cre/loxP and Flp/FRT, respectively, all from Gene Bridges, Heidelberg, Germany). The oligonucleotides used for PCR amplification are listed in supplementary material Table S2.

### Generation of conditional knockout mice

The targeting construct was linearized by *Sal*I (Thermo Fisher Scientific, Waltham, MA) digestion and electroporated into R1 ES cells (250 V, max. 500 μF, 5.5–6.5 millisecond pulse length) (Nagy et al., 1993). After 9 days of G418 selection, clones were separated and expanded into four 96-well culture plates, two of which were frozen for later expansion of positive clones. The two other replicas were used for genomic DNA isolation, which was subsequently digested using *Nde*I (Thermo Fisher Scientific) and analysed by Southern blot. Positive clones were thawed, injected into E3.5 blastocysts of C57BL/6J mice and transferred into the uterine horns of foster mothers. Male chimeric offspring were mated to C57BL/6 females and the resulting offspring analysed for transmission of the targeted allele (N_1_ generation). Transgene-positive male offspring were mated to Flp-deleter in order to remove the selection cassette and subsequently backcrossed to the C57BL/6 background over 10 generations (N_10_) (Rodríguez et al., 2000). To enable an early embryonic knockout of *Dohh*, mice of the B6.C-Tg(CMV-cre)1Cgn/J strain (Schwenk et al., 1995) were mated to mice of the *Dohh*^+/flox^ genotype. Mice of the resulting Dohh^+/−^ genotype were further mated to individuals of the same phenotype.

### Genotyping using genomic DNA from tail clippings, embryos or culture cells

Samples were digested overnight at 55°C using Proteinase K (Thermo Fisher Scientific, Waltham, MA) according to manufacturer’s instructions. PCR analyses of genomic DNA were performed using 2 μl of lysate in a total volume of 20 μl. The primer sequences are listed in supplementary material Table S2.

### *C. elegans* strains and DIC microscopy

All strains were maintained and raised at 20°C on NGM agar seeded with *Escherichia coli* OP50 (Brenner, 1974). The following mutation was used in this study: *dohh-1(gk398)/mIn1[mIs14 dpy-10(e128)] II*. Nematode strains used in this work were provided by the Caenorhabditis Genetics Center, which is funded by the NIH National Center for Research Resources (NCRR). For microscopic evaluation, worms were placed on 3% agarose pads and anaesthetized in 10 μl M9 containing 5 mM levamisole (Sigma-Aldrich, St Louis, MO) and mounted under a coverslip for observation using a Leica DM-RA microscope equipped with DIC (Nomarski) optics.

### Cell culture

Mouse embryonic fibroblasts (MEFs) were isolated from *Dohh*^+/flox^ × *Dohh*^+/flox^ matings as described (Sun et al., 2007). Using the 3T3 protocol (Todaro et al., 1963), a *Dohh*^flox/flox^ and a *Dohh*^+/+^ cell line were generated. The cells were cultured in DMEM (all cell culture media and additives from Invitrogen, Carlsbad, CA) supplemented with 10% fetal bovine serum, 50 U/ml penicillin, 50 μg/ml streptomycin, 25 μM β-mercaptoethanol, and 4 mM L-glutamine (37°C, 5% CO_2_, humidified atmosphere). 10^6^ cells were seeded on a 10-cm culture dish, maintained at subconfluence and treated with 100 nM 4-OHT (Sigma-Aldrich, St Louis, MO) or 15 μM GC7 (Biosearch Tech., Novato, CA) as indicated in the figures. Cell number and viability were assessed using a ViCell cell counter (Beckman Coulter, Brea, CA).

### Cell cycle analysis

Cell cycle analysis by propidium iodide staining was performed as described (Riccardi and Nicoletti, 2006), followed by flow cytometric analysis (FACSCalibur, BD, Franklin Lakes, NJ).

### Proliferation of 3T3 *Dohh*^flox/flox^; Cre^pos.^ cells compared with 3T3 *Dohh*^flox/flox^; Cre^neg.^ cells

Cells were cultured as described above. Both cell lines (3T3 *Dohh*^flox/flox^; Cre^pos.^ cells and 3T3 *Dohh*^flox/flox^; Cre^neg.^ cells) were seeded and treated with 100 nM 4-OHT for 48 hours. At 96 hours after removal of 4-OHT, cells were seeded at a concentration of 2.5×10^5^ cells/10 ml on a 10-cm dish and counted again 96 hours later using trypan blue exclusion assay. All experiments were performed in triplicate.

### Effect of combination of GC7 and 4-OHT on proliferation of 3T3 *Dohh*^flox/flox^; Cre^pos.^ cells

Cells were cultured as described above. 3T3 *Dohh*^flox/flox^; Cre^pos.^ cells were treated with 100 nM 4-OHT or solvent for 7 days. Afterwards, 4-OHT was removed and cells were seeded at a concentration of 2.5×10^5^ cells/10 ml on a 10-cm dish, treated with 50 μM GC7 and counted 48 hours later using trypan blue exclusion assay. All experiments were performed in triplicate.

### Rescue of *Dohh*-deleted cells by retroviral overexpression of DOHH

Murine (mRNA) *Dohh* was PCR-amplified using cDNA purified from NIH3T3 cells using Phu polymerase (Thermo Fisher Scientific, Waltham, MA). The primer sequences are listed in supplementary material Table S2. The 3′-primer was designed to include an additional FLAG-Tag sequence before the stop codon. Furthermore, a point mutant coding for an inactive *Dohh*-E57A (*Dohh*mut) mutant (Kang et al., 2007) was generated using the Phusion site-directed mutagenesis kit (Finnzymes, Vantaa, FIN). Inserts and the pBabe-hygro plasmid (Addgene number 1765) were digested with *Eco*RI and *Bam*HI restriction enzymes (FastDigest, Thermo Fisher Scientific, Waltham, MA) and ligated using T4 DNA ligase (all according to the manufacturer’s instructions). Ligations were transformed into DH5α, ampicillin-selected overnight and verified by sequencing. Subsequent retroviral transduction of either pBabe-hygro, pBabe-*Dohh*-hygro or pBabe-*Dohh*-K57A-hygro plasmids into 3T3 *Dohh*^flox/flox^; Cre^pos.^ cells were performed as described below. Afterwards, stable transduced cells were selected using hygromycin. In order to measure proliferation rate, 3T3 *Dohh*^flox/flox^; Cre^pos.^ *Dohh*-hygro, 3T3 *Dohh*^flox/flox^; Cre^pos.^ *Dohh*-E57A-hygro or 3T3 *Dohh*^flox/flox^; Cre^pos.^ empty-hygro cells were preincubated with 100 nM 4-OHT for 7 days to knock out *Dohh*. Afterwards, cells were seeded at a concentration of 2.5×10^5^ cells/10 ml on a 10-cm culture dish and counted 96 hours later using trypan blue exclusion assay. All experiments were performed in triplicate.

### Cloning of Cre expression plasmid

Tamoxifen-inducible Cre recombinase was PCR-amplified using genomic DNA purified from tail biopsies of the CAG-CreESR1 mouse strain using Phu polymerase (Thermo Fisher Scientific, Waltham, MA). The primer sequences are listed in supplementary material Table S2. Inserts and the pMSCV puro plasmid were digested with the appropriate FastDigest restriction enzymes (Thermo Fisher Scientific, Waltham, MA) and ligated using T4 DNA ligase (all according to manufacturer’s instructions). Ligations were transformed into DH5α, ampicillin-selected overnight and verified by sequencing.

### Retroviral transduction

Ecotropic retroviruses for the transduction of *Dohh*^flox/flox^ cells were obtained by transient calcium-phosphate-mediated transfection of the retroviral vectors into the packaging cell line Phoenix eco (ATCC, Wesel, Germany). *Dohh*^flox/flox^ cells were transduced by adding the filtered, retrovirus-containing supernatant and selected with puromycin as described previously (Balabanov et al., 2011).

### Microarray and real-time qRT-PCR

RNA from 4-OHT-induced (9 days; 100 nM) 3T3 *Dohh*^flox/flox^; Cre^pos.^ and 3T3 *Dohh*^flox/flox^; Cre^neg.^ cells was isolated using TriFast (Peqlab, Erlangen, Germany) according to manufacturer’s instructions. cDNA was prepared by reverse transcription of 1 μg total RNA using oligo(dT) primers and M-MuLV reverse transcriptase (Thermo Fisher Scientific). Real-time RT-PCR was performed using the DyNAmo Flash SYBR Green qPCR Kit (Thermo Fisher Scientific) and QuantiTect Primer Mixes for murine *Gapdh*, *Dohh*, eIF5A2, FZD2, Gremlin-1 and c-Myc (Qiagen, Hilden, Germany) in an Mx3000 real-time thermocycler (Agilent, Santa Clara, CA). All samples were measured in triplicate and normalized against *Gapdh* as reference gene using the 2^−^^ΔΔC(t)^ method (Livak and Schmittgen, 2001). The microarray experiments were performed using Affymetrix mouse genome GeneChips 430 2.0 (Affymetrix, Santa Clara, CA) according to the manufacturer’s protocol starting with 100 ng of total RNA for first strand synthesis. The preparation was performed according to the One Cycle Protocol using the GeneChip 3′ IVT Express Kit from Affymetrix. The arrays were incubated for 16 hours in the Affymetrix Hybridization Oven 640 at 45°C. Washing and staining steps were performed in the Affymetrix Fluidics Station 450 (Command Console 3.0). After washing and staining, the arrays were scanned using the Affymetrix GeneChip Scanner 3000 7G, background corrected and normalized with Affymetrix software (Expression console using RMA). To identify genes that were positively or negatively regulated, we used the TMeV software (4.7.4, www.tm4.org) for hierarchical clustering and *t*-tests (Saeed et al., 2006). A signal-log-ratio of ±0.8 and a *P*-value of 0.05 were used as cut-off values to determine gene lists for further enrichment analysis. Following differential gene expression analysis, we used the obtained lists of regulated genes to perform gene set enrichment and network analysis with Ingenuity Pathway Analysis software (IPA; Ingenuity Systems, Qiagen, Hilden, Germany).

### Senescence-associated β-galactosidase activity

Senescence-associated β-galactosidase activity (SA-β-gal) activity was assessed in cells fixed with 0.25% glutaraldehyde and 2% paraformaldehyde. Cells were incubated at 37°C with a 5-bromo-4-chloro-3-indolyl P3-D-galactoside (X-Gal) staining solution dissolved in PBS containing 1 mM MgCl_2_, pH 5.5 as described (Braig et al., 2005). Per sample, at least 300 randomly selected cells were counted and the number of positive cells was divided by the total number of counted cells.

### Two-dimensional polyacrylamide gel electrophoresis and western blotting

Samples were applied to immobilized pH gradient (IPG) strips (pH 4–7 NL, 24 cm, from GE Healthcare, Munich, Germany) by in-gel rehydration. Per gel, a total amount of 500 μg protein was filled to a volume of 450 μl with rehydration buffer (8 M urea, 2 M thiourea, 2% CHAPS, 15 mM dithiothreitol and 0.5% IPG buffer pH 3–10 NL, from GE Healthcare, Munich, Germany). After rehydration overnight, isoelectric focusing (IEF) and 2D-PAGE were carried out as described previously (Junker et al., 2011).

Western blotting was carried out as described (Dyshlovoy et al., 2012). Refer to supplementary material Table S3 for a list of primary and secondary antibodies used in this study.

### Protein identification by mass spectrometry

Protein spots of interest were excised from the gel manually, followed by an in-gel digest with LysC (200 ng per gel spot) as described previously (Dyshlovoy et al., 2012). For identification of the protein eIF5A and its post-translational modification of lysine to hypusine via the intermediate deoxyhypusine, we performed the measurements on the 4800 MALDI-ToF/ToF™ Analyzer. The spectra were recorded in reflector mode in a mass range from 450 to 1600 Da, with a focus mass of 700 Da, and in a mass range from 875 to 4000 Da, with a focus mass of 2000 Da. The instrument was calibrated with an external calibration using matrix dimers at *m/z* 379.0930 and des-Arg1-Bradykinin at *m/z* 904.4681 for the mass range 450–1,600 or using the Mass Standards Kit for Calibration of AB SCIEX TOF/TOF™ Instruments as default calibration. MALDI-MS/MS analysis was performed for *m/z* 906 and *m/z* 922. The MS and MS/MS spectra were annotated using Data Explorer Software version 4.9 (build 115). Peak detection was carried out with following parameters: valley to baseline; % centroid 50; signal-to-noise threshold 3; noise window width (*m/z*) 250. The fragmentation was proofed by an implemented ion fragmentation calculator with mass tolerance of 0.8 *m/z* and use of monoisotopic masses. A database search of MS measurements was performed online at http://www.matrixscience.com. Peak lists were compared using the SwissProt 2012_08 database mouse taxonomy. Peptide mixtures that yielded a Mowse score of 55 for database results were regarded as positive identification.

### Soft agar assay

Soft agar assays were performed as described recently (Ummanni et al., 2011). In brief, 5×10^3^ *Dohh*^flox/flox^ cells treated with either 4-OHT or GC7 were resuspended in 0.35% agar in complete medium and poured over a solidified layer of 0.5% agar. In the case of GC7 treatment, the drug was added to the top agar. The cells were supplied by weekly addition of several drops of fresh medium. After 2–3 weeks of incubation at 37°C, foci were photographed, stained with 0.5% crystal violet and counted.

### Morphological analysis of mouse tissue

Mice were transcardially perfused with a mixture of 4% paraformaldehyde and 1% glutaraldehyde in 0.1 M phosphate buffer at pH 7.4. Sections (100-μm thick) from spleen were cut with a Vibratom (Leica VT 1000S). The sections were rinsed three times in 0.1 M sodium cacodylate buffer (pH 7.2–7.4) and osmicated using 1% osmium tetroxide in cacodylate buffer. Following osmication, the sections were dehydrated using ascending ethyl alcohol concentration steps, followed by two rinses in propylene oxide. Infiltration of the embedding medium was performed by immersing the pieces in a 1:1 mixture of propylene oxide and Epon and finally in neat Epon and hardened at 60°C. Semi-thin sections (0.5 μm) were prepared for light microscopy, mounted on glass slides and stained for 1 minute with 1% Toluidine Blue.

### Characterization of peripheral blood, bone marrow and spleen

Young (2 months old) and old (ten months old) wild-type and heterozygous *Dohh*+/− mice were euthanized by CO_2_ inhalation in accordance with regulations of the veterinary office of the canton Zurich. Peripheral blood was obtained by cardiac puncture and analysed on an ADVIA 2120 automated hemocytometer (Siemens Healthcare Diagnostics, Germany). One hind leg (femur and tibia) and spleen were isolated. Single cell suspensions were prepared by either flushing out bone marrow cells using FACS buffer (PBS containing 2% FCS and 2.5 mM EDTA) or by gently dissociating spleens between two frosted glass slides, followed by red blood cell lysis using ammonium chloride solution. For flow cytometric analysis of major myeloid and lymphoid compartments, cells were incubated with fluorescent dye-labeled monoclonal antibodies (see supplementary material Table S3) for 25 minutes on ice in the dark. After repeated wash steps, cells were resuspended in FACS buffer containing 2 μg/ml Hoechst33324 (Invitrogen, USA) to exclude dead cells. Samples were analysed on a FACS Canto II flow cytometer (Becton Dickinson, USA) and the data analysed using FlowJo software (TreeStar, USA).

### Polysome profiling

Cells were tamoxifen-induced using 100 nM 4-OHT for 8–10 days or control-treated in parallel. Prior to collection, cells were treated with 50 μg/ml cycloheximide (CHX) for 30 minutes at 37°C unless otherwise indicated. Cells were collected in polysome lysis buffer (20 mM Tris, 10 mM MgCl_2_, 100 mM NaCl, 0.4% NP-40, Roche Complete Protease Inhibitor, 100 U/ml RNasin and 50 μg/ml CHX in CHX-treated samples), incubated on ice for 20 minutes, and centrifuged at 8000 ***g*** for 10 minutes to pellet cell fragments. Protein content of the lysate was determined (Bio-Rad Protein Assay) and equal amounts of total protein were loaded onto 17.5–50% sucrose density gradients (20 mM Tris, 5 mM MgCl_2_, 100 mM NaCl and 50 μg/ml CHX in CHX-treated samples) formed by a Gradient Master (BioComp) and ultracentrifuged for 2.5 hours in an SW40Ti Rotor (Beckman Coulter) at 35,000 rpm. Gradients were fractionated by a Piston Gradient Fractionator (BioComp). Curves were plotted on the same *X*/*Y* axis. Areas under the curve from disome to the end of the graph and monosome alone were measured using ImageJ pixel area measurement software.

### Metabolic labelling to measure protein synthesis rates

On the day of metabolic labelling, cells were washed in pre-warmed 1× PBS. After the wash, 4 ml pre-warmed methionine- and cysteine-free medium were added and cells were incubated for 1 hour at 37°C. As a negative control, CHX was added at a final concentration of 300 nM to one plate each of *Dohh*^flox/flox^;Cre^neg.^, *Dohh*^flox/flox^;Cre^pos.^ or *Dohh*^flox/flox^;Cre^pos.^ + 4-OHT. Click-IT^®^ AHA (Invitrogen) was added to 100 μM final concentration and cells were further incubated for 2 hours at 37°C. Plates were then chilled on ice, 3 ml of medium was removed and cells were harvested using a cell scraper and transferred to microcentrifuge tubes. Cells were washed three times in ice-cold 1× PBS and lysed in 25 μl ice-cold lysis buffer in the presence of protease and phosphatase inhibitors. Samples were sonicated and centrifuged. Then, 5 μl H_2_O and 50 μl 2× Click-IT^®^ Reaction Buffer, 5 μl Component C (CuSO_4_) and 5 μl fresh Click-IT^®^ Reaction Buffer Additive 1 were added and the samples incubated for 3 minutes at room temperature. After addition of 10 μl Click-IT^®^ Reaction Buffer Additive 2, samples were incubated 20 minutes at room temperature. Proteins were subsequently precipitated and pellets were air-dried for 15 minutes. Proteins were re-solubilized in 25 μl 1× LDS NuPAGE loading buffer and incubated for 10 minutes at 70°C. Proteins were separated on a NuPAGE Novex 4–12% BisTris-Acrylamide gel and the TAMRA signal was detected and quantified using a Fujifilm Fluorescent Image Analyzer FLA-9000. The gel was stained using SYPRO Ruby (Life Technologies) according to the manufacturer’s instructions for detection and quantification of total protein content.

### Statistics

The statistical tests used in each analysis are stated in the corresponding figure legends. Statistical analyses were performed using GraphPad Prism (GraphPad Software Inc, San Diego, CA). *P*-values are depicted using asterisks, with *P<0.05, **P<0.01 and ***P<0.001. Error bars in all figures represent the s.e.m. or s.d.

## Supplementary Material

Supplementary Material
