# Supplementary material for: A novel mouse model for inhibition of DOHH-mediated hypusine modification reveals a crucial function in embryonic development, proliferation and oncogenic transformation
Source: Dis Model Mech. 2014 May 15;7(8):963–76. doi: 10.1242/dmm.014449 (PMC4107325; doi:10.1242/dmm.014449)
Supplement: Supplementary Material [file supp_7_8_963__index.html]

A novel mouse model for inhibition of DOHH-mediated hypusine modification reveals a crucial function in embryonic development, proliferation and oncogenic transformation — Supplementary Material 

# A novel mouse model for inhibition of DOHH-mediated hypusine modification reveals a crucial function in embryonic development, proliferation and oncogenic transformation

## DMM014449 Supplementary Material

**Files in this Data Supplement:**

- **Supplementary Material**
