# Supplementary material for: A novel mouse model for inhibition of DOHH-mediated hypusine modification reveals a crucial function in embryonic development, proliferation and oncogenic transformation
Source: Dis Model Mech. 2014 May 15;7(8):963–76. doi: 10.1242/dmm.014449 (PMC4107325; doi:10.1242/dmm.014449)
Supplement: Supplementary Material [file supp_7.8.963_DMM014449.pdf]

Supplementary material

Supplementary figures and legends

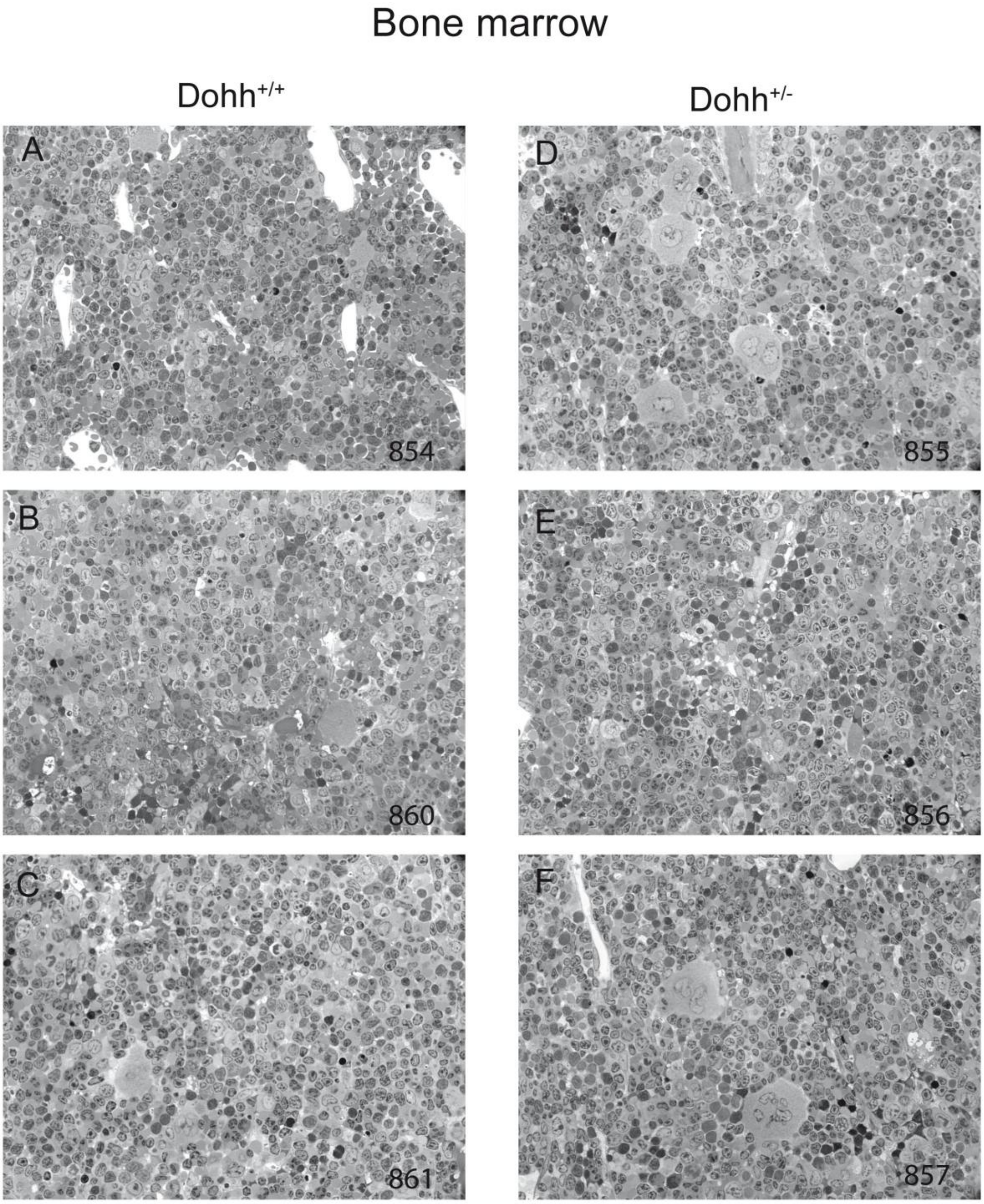

Fig. S1A-F

Fig. S1. Characterization of bone marrow. (A-C) Representative photomicrographs of bone marrow from three wild type *Dohh*$^{+/+}$ mice. **(D-F)** Representative photomicrographs of bone marrow from three heterozygous *Dohh*$^{+/-}$ mice.

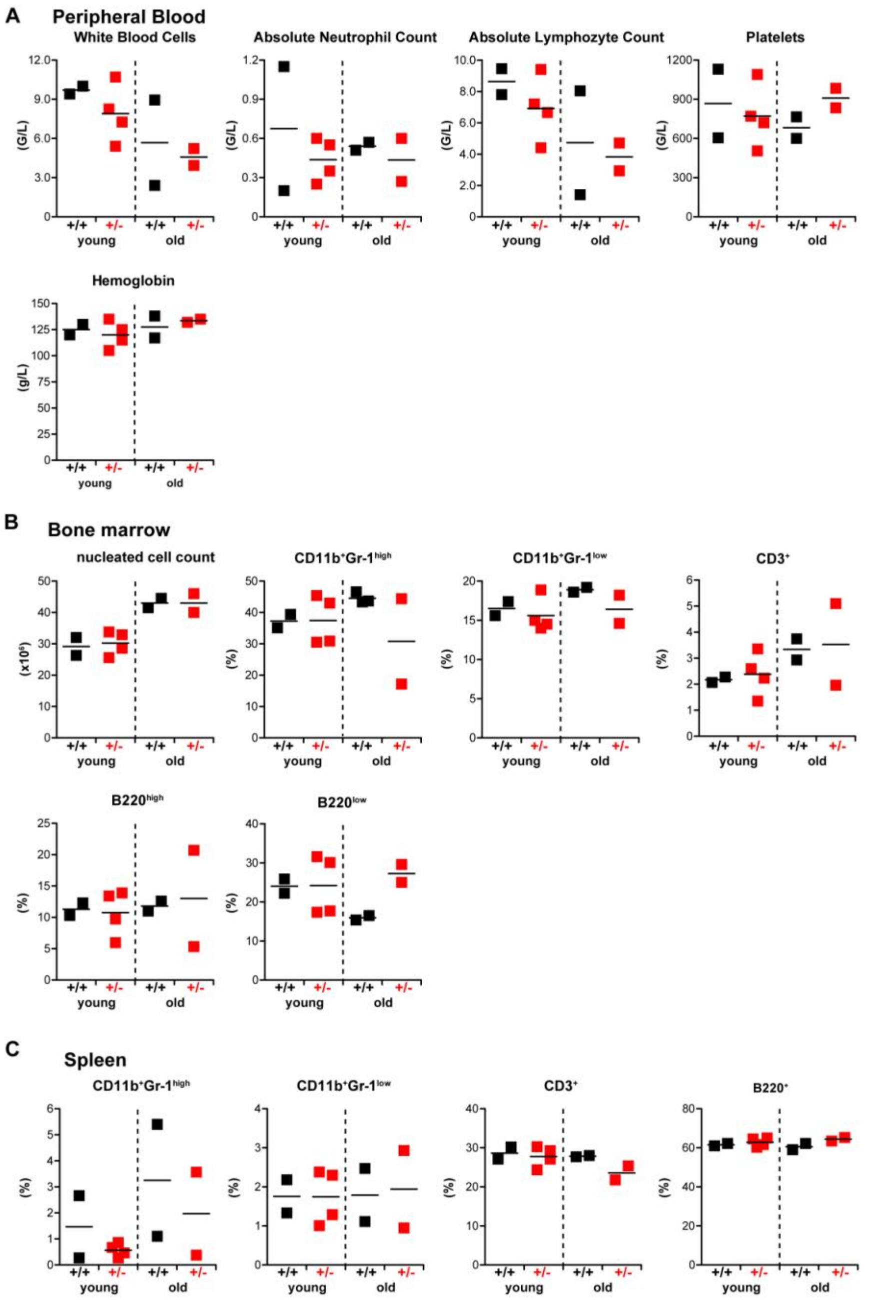

Fig. S2A-C

Fig. S2. Characterization of hematopoiesis. (A) Comprehensive summary of peripheral blood cell counts using ADVIA 2120. **(B)** Comprehensive summary of bone marrow by flow cytometry analysis using CD11b, Gr-1, CD3 and B220 fluorescence labeled antibodies. Nucleated cell count has been analyzed using ADVIA 2120**. (C)** Comprehensive summary of spleen cells by flow cytometry analysis using CD11b, Gr-1, CD3 and B220 fluorescence labeled antibodies. For the analysis, eight weeks old young and 11 month old adult *Dohh*$^{+/+}$ (n=2 for young and n=2 for old) as well as *Dohh*$^{+/-}$ (n=4 for young and n=2 for old) mice have been used.

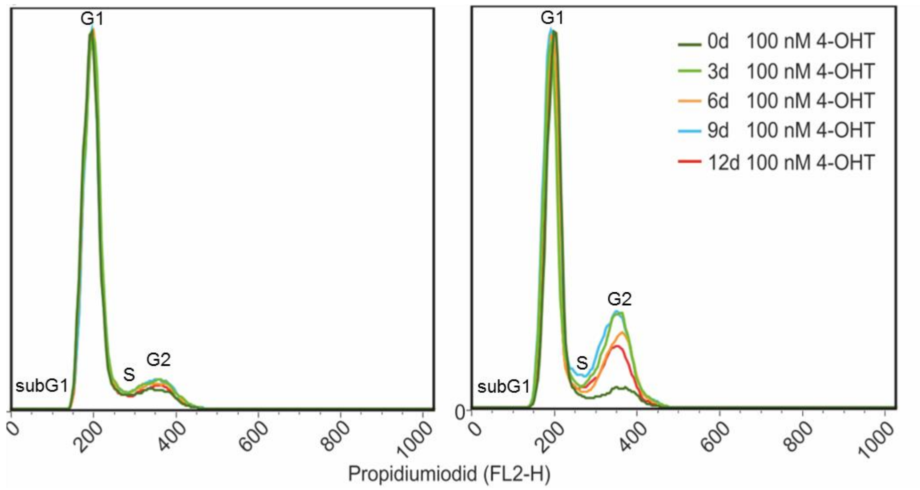

Fig. S3. Deletion of *Dohh* affects cell cycle. Cell cycle analysis was performed using PI-staining of 3T3 *Dohh*$^{flox/flox}$; Cre$^{pos.}$ cells after 4-OHT induction at indicated time points, compared to 4-OHT treated 3T3 *Dohh*$^{flox/flox}$; Cre$^{neg.}$ control cells.

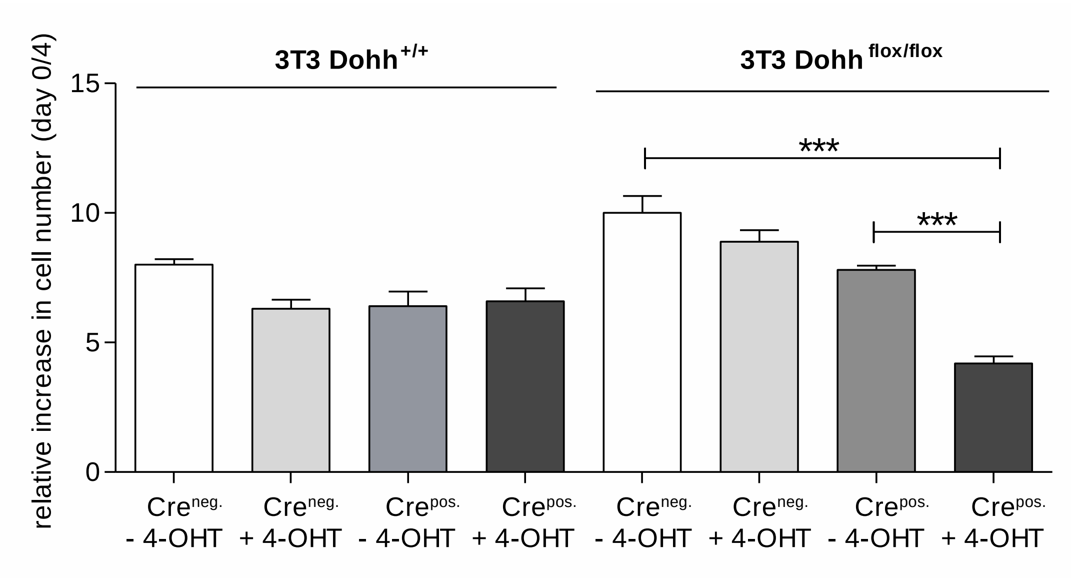

Fig. S4. Additive effects of *Dohh* deletion and GC7 treatment. 3T3 *Dohh*$^{+/+}$; Cre$^{pos.}$ cells and 3T3 *Dohh*$^{flox/flox}$; Cre$^{pos.}$ cells as well as their Cre$^{neg.}$ counterparts were preincubated for seven days with 100 nM 4-OHT to induce *Dohh* KO. Cells were seeded (day 0) and counted 96h later using trypan blue exclusion assay. Depicted is the relative increase in cell number compared to day 0. All experiments were performed in triplicates. Level of significance was analyzed by the student´s t-test using GraphPad Prism (GraphPad Software Inc, San Diego, CA). Statistical significances were marked with asterisk (*** $p< 0.001$; ** $p<0.01$).

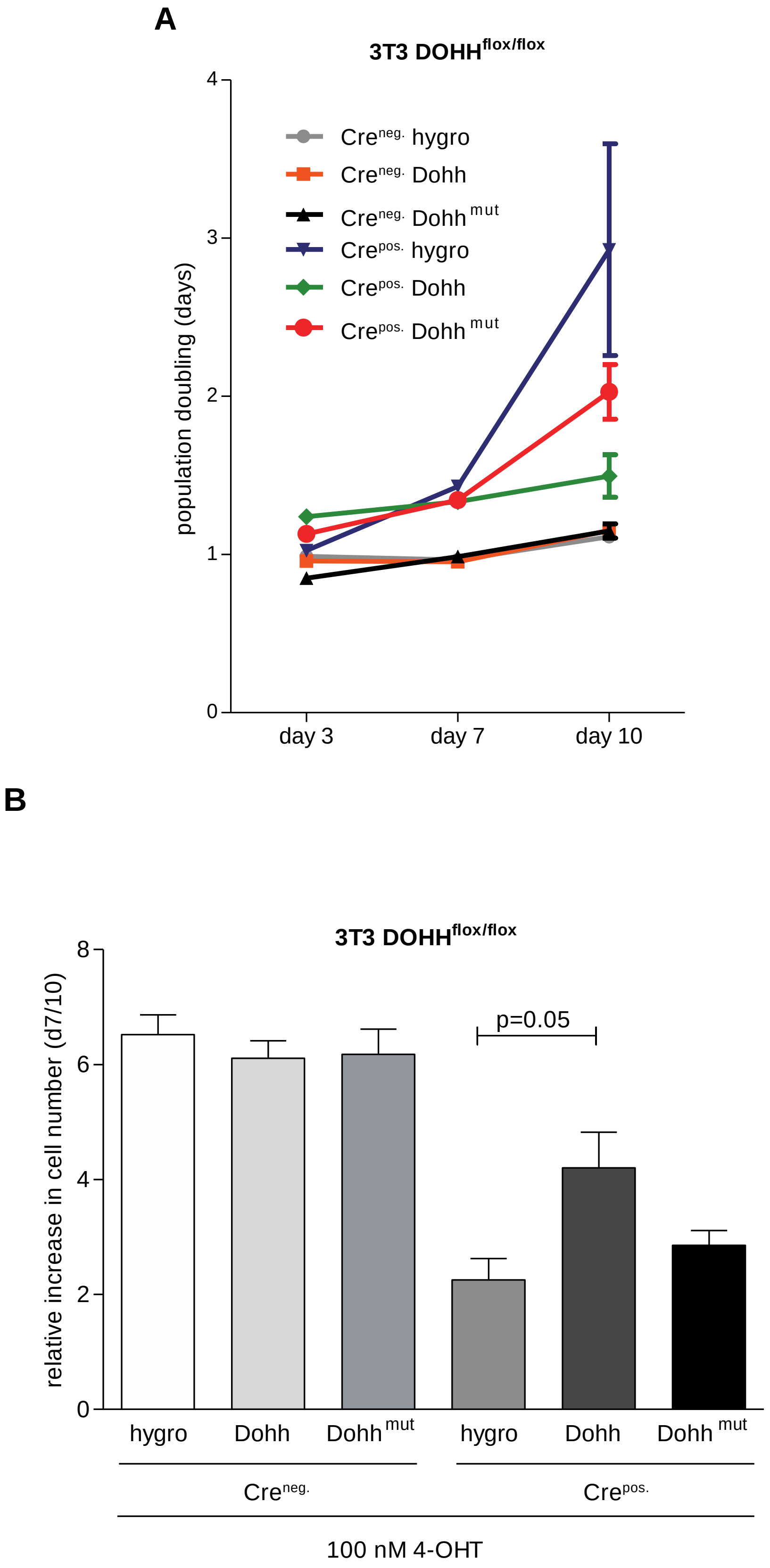

Fig. S5. Ectopic overexpression of *Dohh* partially compensates for *Dohh* deletion. *Dohh*$^{flox/flox}$; Cre$^{pos.}$ or *Dohh*$^{flox/flox}$; Cre$^{neg.}$ cells retrovirally overexpressing *Dohh*, *Dohh*^{mut} or empty vector (hygro) control were preincubated with 100 nM 4-OHT for seven days to induce *Dohh* KO and proliferation was assessed using trypan blue exclusion assay (day 0-7). After

release of 4-OHT, cell number was reassessed the following 96h (day 7-10). **(A)** Population doubling curves depict increased proliferation capacity after introduction of *Dohh* in *Dohh* deleted cells compared to control cells. Effect of mutant *Dohh* was less pronounced. **(B)** Same experiment as in (A). Depicted is the relative increase in cell number from day 7 to day 10. All experiments were performed in triplicates. Level of significance was analyzed by the student´s t-test using GraphPad Prism (GraphPad Software Inc, San Diego, CA). Statistical significances were marked with asterisk.

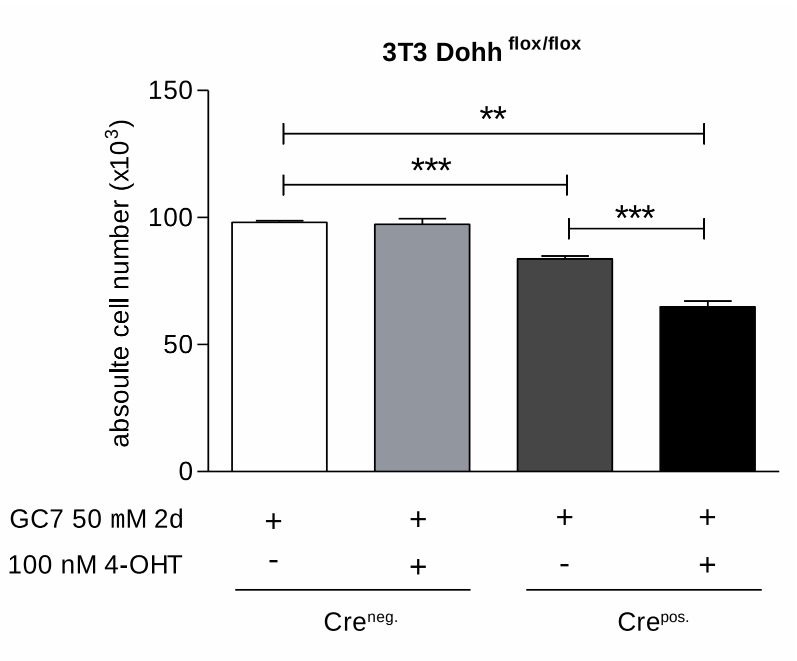

Fig. S6. Additive effects of *Dohh* deletion and GC7 treatment. 3T3 *Dohh*$^{flox/flox}$; Cre$^{pos.}$ cells were preincubated for seven days with 100 nM 4-OHT to induce *Dohh* KO. Afterwards cells were treated with 50 mM GC7 for 48h and counted using trypan blue exclusion assay. All experiments were performed in triplicates. Level of significance was analyzed student´s t-test using GraphPad Prism (GraphPad Software Inc, San Diego, CA). Statistical significances were marked with asterisk (*** $p< 0.001$; ** $p<0.01$).

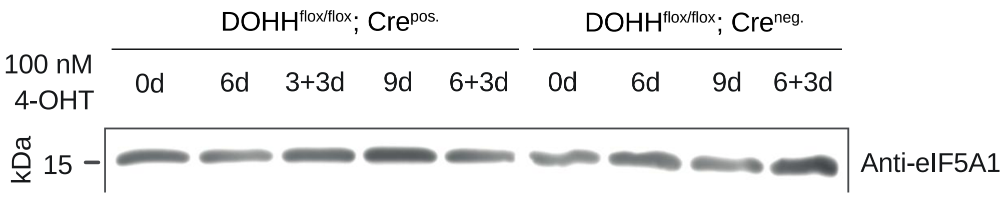

Fig. S7. Effect of *Dohh* deletion on eIF5A1 protein expression. 3T3 *Dohh*$^{flox/flox}$; Cre$^{neg.}$ and 3T3 *Dohh*$^{flox/flox}$; Cre$^{pos.}$ cells were treated with 100 nM 4-OHT for different time points as indicated and eIF5A1 expression was measured by Western blot. See Table S3 for antibody information and Fig. 3 for loading control in the main manuscript.

Supplementary tables

Download Table S1.

Table S2. Oligonucleotides and PCR primer used in this study.

**Oligos used for cloning of the *Dohh* targeting vector**	
Dohh-Step1-for	CAGGTGGTGGTGGCGCATGCCTTTAATCCCAGCACTTGGATGG ATGTGGAGTTAACAATTAACCCTCACTAAAGGGCG
Dohh-Step1-rev	CCGGGATGCTGAGGCAGGAGGACCCCTGAGCTCCAGGCCACA CCGGGATGCTGAGGCAGGAGGACCCCTGAGCTCCAGGCCACA
Dohh -Step2-for	CCTGACTGCCACACTTGGTTGTGTGCACGTGACAGCGTGGACG TCCCATCGCTCTCCTGAGTAGGACAAATC
Dohh -Step2-rev	AACCACCAACGGGAATCTGAGACTGAGATAGCAGCATGTGTT GAACACCAGTCGACTCACAGCTTGTCTGTAAGCGGATG
Dohh -Step3-for	AGGTGCCCCACACAAACCCTGGGGACTTTACCTGGCTGCCTAA CCATAGTTAATACGACTCACTATAGGG
Dohh -Step3-rev	AGCAGGAAACAGTGGGCACATAGGGTCCCAAGGAAGGGACAC ACCAAGACCAATTAACCCTCACTAAAGG
**PCR primer for genotyping**	
Dohh genot. 1 for	AATATCACGGGTAGCCAACG
Dohh genot. 1 rev	TGAATGAACTGCAGGACGAG
Dohh genot. 2+4 for	GGCCACTCTTTGAGCGATAC
Dohh genot. 2+3 rev	CCATGGGACTCTCAGTGGTC
Dohh genot. 3 for	GGAGGGGACGGATAAAGTGT
Dohh genot. 4 rev	GGCCGCATAACTTCGTATAA
Cre genot. for	GCGGTCTGGCAGTAAAAACTATC
Cre genot. rev	GTGAAACAGCATTGCTGTCACTT
**Cloning primers**	
CreEsr1 pMSCV for	CTAGGTCGACGCCACCATGTCCAATTTACTGACCG
CreEsr1 pMSCV rev	CTAGGAATTCTCAGATCGTGTTGGGG
Dohh southern for	CTCGAGCAAGGCAGGAAGAGGAAGTG
Dohh southern rev	CTCGAGGAGGCTGCAAAGGACAAGAC
Dohh pBabe hygro for	CTAG GGATCC GCCACC ATGGTAACGGAGCAAGAGAT
Dohh pBabe hygro rev	CTAG GAATTC CTA CTTGTCGTCATCGTCTTTGTAGTC GGGTGGTGGCCGCAGGCGCT

Table S3. Antibodies used in this study.

Antibody	Host	Supplier	Dilution
Anti-DOHH*	Rabbit	Eurogentec, Serain, B	1:1.000
Anti-eIF5A1	Rabbit	Novus Biol., Littleton, CO, (EP526Y)	1:5.000
Anti-GAPDH	Mouse	Millipore, Billerica, MA (AB2302)	1:1.000
Anti-Rabbit-HRP	Goat	Cell Signaling, Boston, MA (#7074)	1:10.000
Anti-Mouse-HRP	Sheep	GE Healthcare, Piscataway, NJ (NA931)	1:10.000
Anti-Mouse-CD3ε	Rat	eBioscience, San Diego, CA (145-2C11)	1:100
Anti-Mouse-B220	Rat	eBioscience, San Diego, CA (RA3-6B2)	1:50
Anti-Mouse-Gr-1	Rat	eBioscience, San Diego, CA (RB6-8C5)	1:100
Anti-Mouse-CD11b	Rat	eBioscience, San Diego, CA (M1/70)	1:1,000

*Not commercially available. Generated by immunisation of 2 rabbits with *N*-QDFQYADGLERLRPPP-*C* by Eurogentec (Seraing, BELGIUM).
